# Reliability and validity of the Japanese version of the INSPIRE measure of staff support for personal recovery in community mental health service users in Japan

**DOI:** 10.1186/s12888-020-2467-y

**Published:** 2020-02-07

**Authors:** Risa Kotake, Akiko Kanehara, Yuki Miyamoto, Yousuke Kumakura, Utako Sawada, Ayumi Takano, Rie Chiba, Makoto Ogawa, Shinsuke Kondo, Kiyoto Kasai, Norito Kawakami

**Affiliations:** 1 Department of Psychiatric Nursing, Graduate School of Medicine, The University of Tokyo, 7-3-1 Hongo, Bunkyo, Tokyo, 113-0033 Japan; 2 Department of Neuropsychiatry, Graduate School of Medicine, The University of Tokyo, 7-3-1 Hongo, Bunkyo, Tokyo, 113-8655 Japan; 3 Department of Mental Health, Graduate School of Medicine, The University of Tokyo, 7-3-1 Hongo, Bunkyo, Tokyo, 113-0033 Japan; 4 Department of Mental Health and Psychiatric Nursing, Tokyo Medical and Dental University, 1-5-45 Yushima, Bunkyo, Tokyo, 113-8510 Japan; 5 Department of Nursing, Graduate School of Health Sciences, Kobe University, 7-10-2, Tomogaoka, Suma-ku, Kobe, Hyogo 654-0142 Japan; 6 National Institute of Mental Health, National Center of Neurology and Psychiatry, 4-1-1 Ogawahigashi, Kodaira, Tokyo, 187-8553 Japan; 7 Department of Neuropsychiatry, The University of Tokyo Hospital, 7-3-1 Hongo, Bunkyo, Tokyo, 113-8655 Japan; 8 The International Research Center for Neurointelligence (WPI-IRCN), The University of Tokyo Institutes for Advanced Study (UTIAS), 7-3-1 Hongo, Bunkyo, Tokyo, 113-0033 Japan

**Keywords:** Measurement, Mental illness, Personal recovery, Reliability, Validity, Community, Mental health services

## Abstract

**Background:**

Supporting personal recovery in people with mental health difficulties is central to mental health services. This study aimed to develop the Japanese version of INSPIRE and Brief INSPIRE measure of staff support for personal recovery and to evaluate its reliability and validity.

**Methods:**

A questionnaire survey was conducted from October to December 2015. The authors asked users to participate in the survey of 14 community mental health services in the Kanto region of Japan. The service users completed self-administered questionnaires that include the Japanese version of INSPIRE, the Recovery Assessment Scale, the Client Satisfaction Questionnaire, the patient version of the Scale to Assess Therapeutic Relationship in Community Mental Health care and the Short Form Health Survey. Internal consistency was assessed using Cronbach’s alpha coefficient, and test-retest reliability was assessed using the intraclass correlation coefficient (ICC) and weighted kappa. Convergent validity was examined by assessing correlation with other scales. Factor validity was evaluated by exploratory factor analysis (EFA) with generalized least-squares mean and oblimin rotation. In addition, confirmatory factor analysis was used to check the fitness of the factor structure models derived from the EFA.

**Results:**

A total of 195 out of 212 users gave written informed consent and participated in the study. Data from 190 respondents were analyzed (response rate 89.6%). INSPIRE, Brief INSPIRE, and the subscales all showed Cronbach’s alpha coefficient over 0.78. ICC and weighted kappa derived more than 0.92 for subscales and Brief INSPIRE. These numerical values indicated good reliability. The convergent validity of Brief INSPIRE and the subscales was significantly positively correlated with the other scales. Different from the previous study, the factor structure was extracted using EFA. Both factor structures were checked by CFA, but the degree of fitness index was not good in either. Therefore, the factor analysis did not show goodness of fit.

**Conclusions:**

This study found the Japanese version of INSPIRE and Brief INSPIRE to be reliable and valid for use among community mental health service users in Japan.

## Background

In 1998, recovery began to be introduced in Japan [1–3]. Supporting personal recovery is a key aim for mental health services in many countries, including Japan [4, 5]. In 2004, the Headquarters for Mental Health and Welfare of the Ministry of Health, Labour and Welfare proposed a conversion from hospital-based medical treatment to community-based care as a reform vision for mental health welfare [6]. Various community-based mental health services are currently being provided in Japan (e.g., day care, psychiatric home-visit nursing, transition support for employment) [7, 8]. Around the same time, a program of self-empowerment, *Tojisya Kenkyu* was initiated at Urakawa Bethel House in Japan [9]. Additionally, programs aimed toward recovery similar to those developed in the west, such as Assertive Community Treatment, Wellness Recovery Action Plan, Illness Management and Recovery, and Recovery College are widely practiced in Japan [10–14]. For the evaluation of practice, the psychometric properties of the Japanese version of recovery-related scales in which service users measure their personal recovery and service providers measure their recovery knowledge and attitude have been verified [15–20]. As described above, programs including personal recovery are being used. There are teaching materials and lectures that include the idea of personal recovery, such as Assertive Community Treatment training for multi-disciplinary, psychiatric nursing process training using the strength model, and lectures on recovery-oriented services by service users and peer staff [21–23]. However, there is no specialized training program for mental health practitioners to support service users’ personal recovery in Japan. Although there are various services, the recovery orientation of the service has not been evaluated.

CHIME was developed as a conceptual framework of personal recovery consisting of five factors: connectedness; hope and optimism about the future; identity; meaning in life; and empowerment (giving the acronym CHIME) [24]. Importantly, it is valuable to evaluate mental health services from a service user’s point of view [25]. Thus, INSPIRE was developed and is the only measurement tool that fits well with CHIME, is sufficiently reliable and validated, and evaluates the recovery orientation of the service by the service user [26–28].

This study aimed to examine the internal consistency, reliability, test-retest reliability, and convergent validity of the Japanese version of INSPIRE and Brief INSPIRE, and the construct validity (including the factor-based validity) of INSPIRE for users of community mental health services in Japan.

## Methods

The aims and procedures of this study were approved by the Ethical Committee of the Graduate School of Medicine at the University of Tokyo in Japan (submission #10890-(1)).

We explained verbally and in writing the aims, procedures, the voluntary nature of participation, anonymity, and assurance that there was no disadvantage in non-participation. Written informed consent was obtained from all participants.

### Participants

We sent requests to organizations in the Kanto area between August and November 2015. Organizations were selected via opportunistic sampling. A questionnaire survey was given to users with mental health difficulties at 14 community mental health service centers in the Kanto region of Japan. The services offered included rehabilitation, employment transition support, support for continuous employment, and community activity support centers. A self-administered questionnaire was provided to service users in their center, and questions covered the level of support they receive from mental health care workers. To maintain confidentiality, workers in the center did not collect the questionnaires; instead, the researcher collected the completed questionnaire directly, and/or gave the participant a stamped return envelope and instructed them to send back the questionnaire to the researcher. Data was collected from October to December 2015.

A service user met the following inclusion criteria during the survey period: (1) use of community mental health services, (2) age 18 years or older. Before explaining the request for participation in the study, we asked the service center’s staff whether there were users who felt burdened by the explanation. For example, on the day of study, it was determined that users who were over 18 years of age and at the service center, but who were in the resting room were not asked to participate in the study. The first to fifth authors brought questionnaires to the service centers and explained this study. After written informed consent was obtained, the questionnaire was answered by the participants themselves. The directors of the two centers agreed to cooperate in the re-test to verify the test-retest reliability. We asked users at the two centers to fill out the questionnaire 2 weeks after the first response.

### Development of the Japanese version of INSPIRE

There are two versions of INSPIRE: a 27-item full version (INSPIRE) and a 5-item short version (Brief INSPIRE). INSPIRE is a 27-item assessment of a service user’s experiences of the professional support they receive in their recovery [27]. INSPIRE is comprised of two subscales: a 20-item support subscale and a 7-item relationship subscale. The items in the support subscale include five domains: connectedness (items S1–S4), hope (items S5–S8), identity (items S9–S12), meaning and purpose (items S13–S16), and empowerment (items S17–S20). Support items, e.g., “I feel supported by other people,” are first rated as to whether the individual considers it important for their recovery (yes/no). For the items that are important (i.e., yes), the amount of support that they received from a mental health worker is then rated on a 5-point Likert scale, ranging from 0 (not at all) to 4 (very much). Relationship subscale items, e.g., “I feel listened to by my caseworker,” are rated on a 5-point Likert scale, ranging from 0 (strongly disagree) to 4 (strongly agree). No total score is given for INSPIRE; instead, a score is calculated for each subscale, with 20 items for support, and 7 items for relationship. The support subscale can be calculated when at least one item is rated, and the relationship subscale only when all 7 items are done. Scoring for both subscales comprises the mean of all the item ratings and is converted to a percentage, ranging from 0 to 100. Higher scores for the support subscale indicate more support, and higher relationship subscale scores indicate greater helpfulness for personal recovery.

Brief INSPIRE is a 5-item tool used to assess the level of support for recovery provided by a mental health care worker. Five items are selected from each of the different domains (connectedness, hope, identity, meaning and purpose, and empowerment). Unlike INSPIRE, Brief INSPIRE does not ask if each item is important to the respondent and can be calculated only when all items are answered. Both versions of INSPIRE were found to be valid and reliable in the UK [27].

The English version of the 27-item full version was translated into Japanese, in accordance with guidelines for the translation and adaptation of psychometric scales [29], and was done in five steps. (1) Forward translation: after permission to translate and use of INSPIRE was obtained from the original authors. Three of the researchers carried out independent translations of INSPIRE from English to Japanese. (2) Reconciliation: 11 mental health professionals (nurses, psychiatrists, and social workers) who are also mental health researchers, reached a consensus on a draft Japanese translation of INSPIRE that best reflected the literal and conceptual content of the original INSPIRE. (3) Back-translation: a professional translator, a native English speaker, who did not know about the original English version of INSPIRE, did a back-translation of the Japanese version into English. (4) The back-translation was reviewed and harmonized: the original INSPIRE developer and the authors reviewed the back-translations against the source instrument and ensured the translation was conceptually equivalent to the original. Additionally, the original INSPIRE developer suggested the service user rate the person they see most often or have the closest working relationship with, instead of naming a particular worker, because most mental health service users in Japan do not have a dedicated provider. We adopted this suggestion for improved ease of use in the Japanese population. (5) Cognitive debriefing and finalization: two people who were using community mental health services in Japan tested INSPIRE, and the research team confirmed the level of comprehensibility and cognitive equivalence of the translation. The wording of INSPIRE is deliberately generic [27]. Similarly, the Japanese version of INSPIRE was translated to be simple and comprehensible, so as to enhance the usability of INSPIRE across other services in all processes (Additional file 1).

### Measures

#### Recovery assessment scale

Mental health recovery was assessed using the Recovery Assessment Scale (RAS), a 24-item measure of self-reported recovery. Items such as “I have a desire to succeed” are rated on a 5-point Likert scale, ranging from 1 (strongly disagree) to 5 (strongly agree). The total score ranges 24–120, with higher scores indicating greater recovery [30, 31].

#### Client satisfaction questionnaire

Service user satisfaction was assessed using the 8-item Client Satisfaction Questionnaire (CSQ-8), with higher scores indicating greater satisfaction. Items such as “How would you rate the quality of service you have received,” are rated on a 4-point Likert scale, ranging from 1 (poor) to 4 (excellent). The total score ranges from 8 to 32 [32].

#### Patient version of the scale to assess therapeutic relationships in community mental health care

The relationship between service user and mental health worker was assessed using the patient version of the Scale to Assess Therapeutic Relationships in Community Mental Health Care (STAR-P), a 12-item self-report measure of level of relationship. Items such as “My clinician speaks with me about my personal goals and thoughts about treatment” are rated on a 5-point Likert scale ranging from 0 (never) to 4 (always). The total score ranges from 0 to 48, with higher scores indicating a stronger relationship between the user and the worker [33]. There was no Japanese version of STAR-P. After obtaining permission from the original authors, we replaced the word ‘clinician’ with ‘staff worker’ and translated STAR-P into Japanese.

#### Short form health survey

Quality of life (QOL) was assessed using Short Form 8 (SF-8), an 8-item measure of general aspects of health-related QOL. The SF-8 is comprised of a physical component summary (PCS) and a mental component summary (MCS). Summary scores are calculated in accordance with scoring rules [34], with higher scores indicating better QOL.

#### Demographic variables

Demographic variables included sex, age, marital status, cohabitation, diagnosis (schizophrenia, mood disorder, or other), and length of current service use.

The Japanese versions of RAS, CSQ-8, and SF-8 are reliable and valid [16, 35, 36].

### Statistical analysis

Responses with at least one completed item were included in the analysis. For reliability, the internal consistency for the support subscale as a whole and for each of its five domains, as well as for the relationship subscale and Brief INSPIRE, was assessed using Cronbach’s alpha [37]. Alpha coefficients greater than or equal to 0.70 were considered satisfactory [38].

Test-retest reliability was assessed in a subsample of respondents who were surveyed a second time 2 weeks later. The support subscale and items from Brief INSPIRE with changes in ratings of importance were tested by examining the kappa statistic, and the Likert scale scores were tested by examining the weighted linear kappa for each item. The relationship subscale was examined to calculate a weighted linear kappa for each item. A kappa statistic less than or equal to 0.20 was considered as indicating poor to slight agreement, 0.21–0.40 fair agreement, 0.41–0.60 moderate agreement, 0.61–0.80 substantial agreement, and greater than 0.80 almost perfect agreement [39]. The total scores of the support subscale, the relationship subscale, and Brief INSPIRE were examined to calculate the intraclass correlation coefficient (ICC). An ICC that was greater than 0.80 was considered as indicating excellent agreement [40].

The convergent validity of the support subscale was assessed by correlation with the CSQ-8J and RAS, the relationship subscale by correlation with STAR-P and RAS, and Brief INSPIRE by correlation with CSQ-8J, STAR-P, and RAS using the Pearson product-moment correlation [41]. Pearson’s correlations were classified as poor (≤ 0.40), moderate (0.40–0.70), or strong (> 0.70).

For further analysis, the factor validity of each of the two INSPIRE subscales was assessed using participant responses that answered yes to all 20 items in the support subscale and all seven items in the relationship subscale. The factor validity of each subscale was assessed using exploratory factor analysis (EFA). The suitability of the data for factor analysis was first examined using the Kaiser-Meyer-Olkin (KMO) measure of sampling adequacy and Bartlett’s test of sphericity, with a *p* value less than 0.05 indicating significance for each subscale. The KMO indicator was then compared with adequacy standards (0.80 < meritorious) [42]. The EFA for each subscale among all the respondents of a Likert scale was performed using generalized least-squares means and an oblimin rotation, which used eigenvalues > 1.00 to determine the number of factors. Because INSPIRE was developed based on a theory, the generalized least-squares mean method was selected [43]. In addition, oblimin rotation was chosen due to the presumption that the five domains of the support subscale are correlated with one another. Confirmatory factor analysis (CFA) was implemented to test the fitness of the data to the factor structure extracted from the EFA. Based on theoretical notions, five underlying factors were expected. A 5-factor model was then defined as model 1, and the EFA extracted in this study model was defined as model 2. Model fit was assessed using a combination of fit indices, including the ratio of χ2 to df (≤ 2), the Goodness of Fit Index (GFI; > 0.95), the Adjusted Goodness of Fit Index (AGFI; > 0.95), the Comparative Fit Index (CFI; > 0.95), the root mean square error of approximation (RMSEA; < 0.07), and the Akaike Information Criterion (AIC), where smaller is better [44, 45]. Statistical analyses except for CFA were conducted using SPSS, version 22.0 for Windows, and CFA was conducted using Amos, version 22. Two-tailed values of *p* less than 0.05 were considered statistically significant.

## Results

### Respondent characteristics

Five of the 195 responses were excluded because there was no response to the INSPIRE questionnaire. The remaining 190 responses were included in the analysis (89.6% of the initial 212 service users). We returned to the centers 2 weeks later and asked 15 users who agreed to fill out the re-test questionnaire. The 10 users who were able to connect to the initial questionnaire were included in the test-retest reliability analysis. The sociodemographic data and average score for each scale of the respondents are shown in Table 1. There were more male than female respondents. The age range was 18–75 years (mean ± SD 42.5 ± 11.5), and more than 70% had never been married. Half of the participants (50%) had received a diagnosis of schizophrenia, and 30% had been diagnosed with a mood disorder. About 65% of participants had used the current service for longer than 1 year.

**Table 1:** Socio-demographic characteristics of the respondents

Variables	*N* = 190	
Sex — no. (%)		
Male	117	(61.6)
Female	73	(38.4)
Missing	0	
Age — average. (SD)		
Age (years)	43	(11.5)
Missing	7	
Marital status — no. (%)		
Never married	147	(77.4)
Currently married	19	(10.0)
Divorced/ widowed	20	(10.5)
Missing	4	(2.1)
Living situation — no. (%)^a^		
Single	50	(26.7)
Parents	105	(56.1)
Sibling	31	(16.6)
Partner	15	(8.0)
Child	10	(5.3)
Other	11	(5.9)
Missing	3	
Diagnosis — no. (%)^a^		
Schizophrenia	100	(53.5)
Mood disorder	57	(30.5)
Development disability	17	(9.1)
Anxiety disorder	15	(8.0)
Other	45	(24.1)
Unknown	11	(5.9)
Missing	3	
Length of use in the current service — average. (SD)		
Months	48	(63)
Missing	12	
Scale scores^b^ — average. (SD)		
INSPIRE Support sub-scale	72.9	(21.7)
Missing	5	
INSPIRE Relationship sub-scale	77.8	(20.9)
Missing	7	
Brief INSPIRE	78.5	(19.3)
Missing	48	
SF8 physical component summary score	48.1	(8.65)
Missing	11	
SF8 mental component summary score	42.7	(8.95)
Missing	11	
STAR-P	33.6	(9.25)
Missing	3	
CSQ8J	23.8	(4.37)
Missing	12	
RAS	82.2	(15.5)
Missing	2	

^a^ Total percentages will exceed 100 because of multiple responses

^b^*SF8* The Short-Form health survey, *STAR-P* Scale To Assess the Therapeutic Relationship in Community Mental Health Care Patient Version, *CSQ-8J* Client Satisfaction Questionnaire, *RAS* The Recovery Assessment Scale

### Descriptions of ratings in INSPIRE

The item-level ratings of the support subscale and the relationship subscale are shown in Table 2 and Table 3, respectively. Four support subscale items (S4, S9, S11, and S12) were rated as not important for recovery by more than 15% of respondents. More nonresponses were found in item S12 (“Having my ethnic/cultural/racial/identity respected”), compared to other items. There were few unanswered items in relationship subscale. Except for two INSPIRE items (S9 and S12), all other items were found to have ceiling effects. Most participants agreed to receive recovery-oriented services from their workers regarding these items, and the item score distribution was skewed disproportionately higher.

**Table 2:** Item-level ratings of the Japanese version of the INSPIRE Support sub-scale (*N* = 190) and factors derived from the Japanese version of the INSPIRE Support sub-scale (*n* = 106^b^): exploratory item factor analysis with generalized least-squares method and oblimin rotation

		Not important		Support							
No.	Items	n	(%)	mean	SD	ceilingeffect	flooreffect	Factor 1	Factor 2	Factor 3	Missing
Domain Connectedness											
^a^S1	Feeling supported by other people	11	5.8	3.43	.90	4.32	2.53	−.021	**.942**	−.108	3
S2	Having positive relationships with other people	11	5.8	3.15	1.02	4.17	2.13	.009	**.710**	.251	2
S3	Having support from other people who use services	18	9.5	3.16	1.03	4.19	2.13	.138	**.369**	**.311**	3
S4	Feeling part of my community	33	17.4	2.97	1.12	4.09	1.85	.276	**.321**	.150	3
Domain Hope											
S5	Feeling hopeful about my future	21	11.1	2.98	1.16	4.14	1.82	.188	.171	**.537**	2
S6	Believing that I can recover	18	9.5	3.12	1.03	4.15	2.09	−.104	−.001	**.836**	6
S7	Feeling motivated to make changes	15	7.9	3.09	1.09	4.18	2.00	.005	.001	**.821**	5
^a^S8	Having hopes and dreams for the future	21	11.1	3.07	1.04	4.11	2.04	**.351**	−.009	**.597**	5
Domain Identity											
S9	Feeling I can deal with stigma	29	15.3	2.57	1.27	3.84	1.31	**.394**	.177	**.335**	5
^a^S10	Feeling good about myself	23	12.1	2.91	1.15	4.05	1.76	.166	.179	**.514**	5
S11	Having my spiritual beliefs respected	30	15.8	2.93	1.12	4.04	1.81	.124	.210	**.569**	6
S12	Having my ethnic/cultural/racial identity respected	29	15.3	2.69	1.16	3.84	1.53	**.448**	−.043	**.338**	10
Domain Meaning and purpose											
S13	Understanding my mental health experience	15	7.9	3.07	1.01	4.08	2.06	**.536**	**.359**	.007	5
^a^S14	Doing things that mean something to me	12	6.3	3.02	1.10	4.12	1.92	**.411**	.238	.208	7
S15	Rebuilding my life after difficult experiences	13	6.8	3.04	1.07	4.11	1.97	**.915**	−.017	−.089	6
S16	Having a good quality of life	12	6.3	3.05	1.06	4.10	1.99	**.945**	−.024	−.103	5
Domain Empowerment											
^a^S17	Feeling in control of my life	18	9.5	2.90	1.16	4.05	1.74	**.483**	.041	.246	7
S18	Being able to manage my mental health	13	6.8	2.98	1.10	4.07	1.88	**.660**	.116	.059	6
S19	Trying new things	20	10.5	3.05	1.02	4.07	2.03	**.540**	−.019	**.421**	3
S20	Building on my strengths	15	7.9	3.15	1.02	4.17	2.13	**.590**	.099	.167	5

Bold figures indicate factor loading > 0.3

^a^ items of Brief INSPIRE (5-items; S1, S8, S10, S14, S17)

^b^ Respondents that answered “Yes” to all the 20 item in support sub-scale

**Table 3:** Item-level ratings of the Japanese version of the INSPIRE Relationship sub-scale (*N* = 190) and factors derived from the Japanese version of the INSPIRE Relationship sub-scale (*n* = 183^a^): exploratory item factor analysis with generalized least-squares method and oblimin rotation

No.	Items	Support mean	SD	ceiling effect	floor effect	Variance explained	KMO score	Missing
R1	I feel listened to by my worker	3.24	1.03	4.27	2.21	59.25	0.87	0
R2	I feel supported by my worker	3.22	1.04	4.25	2.18			1
R3	I feel that my worker takes my hopes and dreams seriously	2.98	1.12	4.10	1.86			3
R4	My worker respects me	3.13	1.02	4.16	2.11			4
R5	My worker treats me as an individual – more than a ‘diagnosis’ or a ‘label’	3.12	1.08	4.20	2.04			2
R6	My worker supports me to make my own decisions	3.09	1.02	4.11	2.06			2
R7	My worker keeps hopeful for me even when I feel at my lowest	3.01	1.06	4.07	1.95			3

^a^Respondents that answered all 7 items in relationship sub-scale

### Reliability of INSPIRE

#### Internal consistency reliability

The Cronbach’s alpha coefficients indicating internal consistency reliability of the five domains were 0.78 (connectedness), 0.88 (hope), 0.86 (identity), 0.88 (meaning and purpose), and 0.86 (empowerment). The total scores for support subscale, relationship subscale, and Brief INSPIRE were 0.96, 0.90, and 0.82, respectively.

#### Test-retest reliability

The ICC for the total scores of the support subscale, relationship subscale, and Brief INSPIRE were 0.95, 0.96, and 0.92, respectively. The kappa statistic of change in rating of importance (yes/no) for the support subscale and Brief INSPIRE were 0.48 and 0.39, respectively. The weighted linear kappa for the support subscale, relationship subscale, and Brief INSPIRE were 0.96, 0.92, and 0.96, respectively.

### Validity of INSPIRE

#### Convergent validity

The total scores of the support subscale, relationship subscale, and Brief INSPIRE were significantly positively correlated with STAR-P, CSQ-8J, and RAS (Table 4).

**Table 4:** Pearson’s correlation coefficients of the Support sub-scale, Relationship sub-scale, Brief INSPIRE with related scales

		Total score of Support	Total score of Relationship	Total score of Brief INSPIRE
Scales^b^	n^a^			
STAR-P score	187		0.80**	0.71**
CSQ-8 J score	178	0.57**		0.55**
RAS score	188	0.49**	0.50**	0.50**

^a^The number of subjects varied because of missing responses for each related scale. ^b^*STAR-P* Scale To Assess the Therapeutic Relationship in Community Mental Health Care Patient Version, *CSQ-8J* Client Satisfaction Questionnaire, *RAS* The Recovery Assessment Scale. ** *p* <  0.01

#### Factor validity

To assess the factor validity of the INSPIRE support subscale, the 106 responses in which yes was given to all 20 items in the support subscale (50.0% of the initial 212) were utilized. Similarly, for the relationship subscale, the 183 responses that answered all 7 items in the relationship subscale (86.3% of the initial 212) were utilized.

For the support subscale, the KMO score was 0.93 and Bartlett’s test of sphericity was significant (χ2 = 1544.57, df = 190, *p* <  0.001), thereby indicating that the factor analysis was appropriate. EFA was conducted among the 106 respondents and yielded three factors based on the criteria of eigenvalues greater than 1.00 (Table 2). Three factors were considered to represent connectedness, hope and internal value, and meaning, purpose, and empowerment in life. Items S3, S8, S9, S12, S13, and S19 had factor loadings greater than 0.30 for two factors (Table 2). CFA showed both models were useful for nested fit (χ2 /df = 1.5 for model 1 and 1.6 for model 2), but other goodness-of-fit indexes were nonoptimal fits for each model. In a comparison of the two models, model 1 was better than model 2 in scores for all indexes, especially for the AIC of model 1, which was smaller than that for model 2 (Table 5). The KMO score for the relationship subscale was 0.87, and Bartlett’s test of sphericity was significant (χ2 = 765.83, df = 21, *p* <  0.001), showing factor analysis was appropriate. EFA was conducted among the 183 respondents, and a 1-factor solution explaining 59.3% of the variance was found. One factor (eigenvalue 4.5) was found (Table 3).

**Table 5:** Results of confirmatory factor analysis: Comparison of goodness-of-fit indexes among Model 1 (five-factor) and Model 2 (three-factor) (*n* = 106^a^)

Model	GFI	AGFI	CFI	RMSEA	AIC	*χ*^*2*^	*df*	p
Five-factor model^b^	0.83	0.77	0.94	0.07	348.01	242.01	157	< 0.001
Three-factor model^c^	0.81	0.75	0.93	0.08	362.19	272.19	165	< 0.001

*GFI* Goodness of Fit Index, *AGFI* Adjusted Goodness of Fit Index, *CFI* Comparative Fit Index, *RMSEA* Root Mean Square Error of Approximation, *AIC* Akaike Information Criterion for model. ^a^Respondents that answered ‘Yes’ to all 20 items in support sub-scale. ^b^model defined based on theoretical notion in the previous study. ^c^model defined by Exploratory item factor analysis in this study.

## Discussion

This study evaluated the reliability and validity of the Japanese version of INSPIRE and Brief INSPIRE among users of community mental health services in Japan. INSPIRE and Brief INSPIRE were found to have high internal consistency reliability, test-retest reliability, and convergent validity, as well as reasonable factor validity, among users of mental health services in Japan. However, the CFA did not show goodness of fit.

### Reliability of the INSPIRE and brief INSPIRE

Internal consistency reliability was found to be acceptable (Cronbach’s alpha variation 0.78–0.96) [38]. These coefficients were found to be good in previous studies [27, 46]. As in a previous study in Sweden, the alpha coefficient of the total score of the support subscale (0.96) was extremely high [46]. This suggests that the support subscale has redundancies and reduces the items [47]. In the development of the original INSPIRE, an item not considered important for recovery by more than half of the respondents was deleted [27]. In this study, as shown in Table 2, more than 80% of respondents indicated important for recovery in all items. Importantly, personal recovery is deeply personal [1]. Thus, we did not delete any items to ensure that the service users could choose what was important for their recovery. The test-retest reliability of the ICC and weighted linear kappa showed superior agreement for the total score of each subscale and Brief INSPIRE [39, 40].

### Validity of INSPIRE and brief INSPIRE

Convergent validity was found to be moderate (Pearson’s correlation 0.49–0.80). These coefficients were similar to those of the previous study in the UK [27].

For the support subscale, the 3-factor structure was extracted using EFA. This 3-factor structure was different from the structure of the theory base in the previous study in which the original INSPIRE was tested [27]. CFA, to test the fitness of the data to the factor structure, revealed both models to be nonoptimal fits. In a comparison of two models, model 1 (theoretical 5-factor model) was better than model 2 (3-factor model). According to the AIC scores, model 1 showed a better fit than model 2. There are two reasons underlying this. First, almost all of the items had ceiling effects. We confirmed the ceiling effect on all items of the INSPIRE, except for S9 and S12. This may indicate bias in the data, and the exclusion of items was considered before factor analysis. However, factor analysis was performed without deleting the items because INSPIRE is a desirable scale for higher scores and the items were created from the CHIME framework [27, 48]. Second, four support subscale items (S4, S9, S11, and S12) were rated as not important for recovery by more than 15% of respondents. These items are included in the domain of connectedness and identity. While Asian cultures focus on building harmonious interdependence with others, American culture tries to maintain independence by paying attention to oneself and through the discovery and expression of one’s own intrinsic inner attributes [49]. Thus, Western and Asian cultures may perceive different relationships and identities. Further studies are needed to investigate the construct of personal recovery in Japan.

In the relationship subscale, the 1-factor structure was extracted using EFA. This was consistent with the structure of the original INSPIRE [27]. Further studies are needed to clarify factor structure validity with a larger number of participants.

### Limitations

There are four main limitations to this study. First, the stability of test-retest reliability is very insufficient because of the small sample size (*n* = 10). Future studies with a large sample size would be needed to clarify test-retest reliability. Second, the convergent validity of the relationship subscale uses the Japanese version of STAR-P, which does not examine psychometric properties. As such, there is a limit to the validity evaluation of the relationship subscale. After completion of this study, the psychometric properties of the Japanese version of STAR were verified in 2019 [50]. Further studies are needed to validate the relationship subscale with the Japanese version of STAR-P that evaluates the psychometric properties. Third, generalization of the findings should be done with caution because the participants were selected only from specific areas and were using specific, limited types of services. Therefore, further research is required and should include diverse services such as visiting care. Fourth, some service centers that cooperated with this study might provide a higher level of support for personal recovery than others, and the participants that responded to the questionnaire did so in accordance with the level of service they received. This could have skewed the data, with overly positive ratings due to bias.

### Research and clinical implications

The research on to how mental health service providers can support personal recovery is developing [51, 52]. The original INSPIRE developer suggested using INSPIRE “as a benchmarking tool for comparison between groups of service users” [27]. INSPIRE will be a valuable tool to determine how a user feels about the services provided. Moreover, the use of INSPIRE by a service provider and user to look back together about the service will be an opportunity to develop better services and relationships. INSPIRE measures the quality of services, and provides a tool to compare Japan’s services internationally. Brief INSPIRE includes the concept of CHIME and can be used as a simple evaluation. However, the brief version does not include individual preference for different types of support or assess the relationship with staff. Therefore, for simple evaluations, we recommend the brief version, and we recommend INSPIRE for more comprehensive evaluations. In Japan, there are previous studies on personal recovery evaluation of service users and recovery knowledge and attitudes of professionals [53–55]. Meanwhile, however, the evaluation of the recovery orientation of the service by the service user has not yet been done. INSPIRE could contribute to a framework in which service users are able to evaluate the recovery-oriented focus of mental health services in Japan. Moreover, INSPIRE can facilitate the development of training programs for mental health practitioners to support the personal recovery of service users in Japan. In addition to evaluating individual mental health services, INSPIRE can be compared with a variety of other mental health services both in Japan and abroad, and thus, can be useful for research to improve the recovery orientation of mental health services.

## Conclusion

This study confirmed the internal consistency, test-retest reliability, and convergent and factor validity of the Japanese version of INSPIRE and Brief INSPIRE among users of community mental health services in Japan (Additional files 1 and 2). INSPIRE and Brief INSPIRE may be useful as patient self-report measures of staff support for personal recovery among Japanese people using community mental health services.

## Supplementary information

**Additional file 1.** Japanese version of INSPIRE.

**Additional file 2.** Japanese version of Brief INSPIRE.

## Data Availability

The datasets generated and analyzed during the current study are not publicly available as permission was not obtained from the participants to publicly share anonymized participant data but are available from the corresponding author on reasonable request.

## Abbreviations

AIC: Akaike Information Criterion
CFA: Confirmatory Factor Analysis
EFA: Exploratory Factor Analysis
ICC: Intraclass Correlation Coefficient
KMO: Kaiser-Meyer-Olkin
MCS: Mental component summary
PCS: Physical component summary
QOL: Quality of life
RAS: Recovery Assessment Scale
