# Supplementary material for: Reliability and validity of the Japanese version of the INSPIRE measure of staff support for personal recovery in community mental health service users in Japan
Source: BMC Psychiatry. 2020 Feb 7;20:51. doi: 10.1186/s12888-020-2467-y (PMC7006071; doi:10.1186/s12888-020-2467-y)
Supplement: Supplementary file 1 — Additional file 1. Japanese version of INSPIRE. [file 12888_2020_2467_MOESM1_ESM.pdf]

INSPIRE

「リカバリー」は色々な意味として語られます。そのひとつは、「満足いく、希望に満ちた人生をおくること」です。
この質問は、担当スタッフが、あなたのリカバリーをどのように支えているかをお聞きするものです。
サポート（支援）の項目では、あなたのリカバリーにとって大切なことについて、そして担当スタッフからそれらについてどれくらい支援されているかをお聞きします。 関係性の項目では、あなたとあなたの担当スタッフとの間の関係についてお聞きします。
（担当のスタッフがいない場合は、あなたがもっともよく接するスタッフ、あるいはよく相談をするスタッフをひとり思い浮かべてお答えください。）
もしもお答えになりたくない質問がありましたら、お答えにならなくてもかまいません。

________________________についてすべての質問にお答えください。
（担当スタッフの名前）

サポート（支援）

それぞれの質問をよんで、それがあなたのリカバリーにとって大切かどうかお考えください。
「大切でない」に〇をつけた場合、次の質問に進んでください。
「大切である」と考えた場合、担当スタッフからどれくらい支援をされているか、灰色の欄のあてはまるところにひとつ〇をつけてください。

私のリカバリーにとって...			担当スタッフから支援されていると感じますか				
1　まわりの人からサポートを受けていると感じること	大切でない	大切である：	そう感じない	あまりそう感じない	どちらでもない	ややそう感じる	そう感じる
2　まわりの人と前向きな関係をもつこと	大切でない	大切である：	そう感じない	あまりそう感じない	どちらでもない	ややそう感じる	そう感じる
3　サービスを利用している他の人たち（メンバーや患者など）からサポートされること	大切でない	大切である：	そう感じない	あまりそう感じない	どちらでもない	ややそう感じる	そう感じる
4　地域の一員であると感じること	大切でない	大切である：	そう感じない	あまりそう感じない	どちらでもない	ややそう感じる	そう感じる
5　将来に希望を感じること	大切でない	大切である：	そう感じない	あまりそう感じない	どちらでもない	ややそう感じる	そう感じる
6　自分はリカバリーできると信じること	大切でない	大切である：	そう感じない	あまりそう感じない	どちらでもない	ややそう感じる	そう感じる

サポート（支援）

私のリカバリーにとって...			担当スタッフから支援されていると感じますか				
7　自分が変わることに前向きであること	大切でない	大切である:	そう感じない	あまりそう感じない	どちらでもない	ややそう感じる	そう感じる
8　将来に夢や希望をもつこと	大切でない	大切である:	そう感じない	あまりそう感じない	どちらでもない	ややそう感じる	そう感じる
9　偏見（へんけん）にうまく対処できること	大切でない	大切である:	そう感じない	あまりそう感じない	どちらでもない	ややそう感じる	そう感じる
10　自分自身をよく思えること	大切でない	大切である:	そう感じない	あまりそう感じない	どちらでもない	ややそう感じる	そう感じる
11　自分の信仰（しんこう）・信念・信条を尊重（そんちょう）されること	大切でない	大切である:	そう感じない	あまりそう感じない	どちらでもない	ややそう感じる	そう感じる
12　文化的背景・民族・人種・国籍（こくせき）を尊重（そんちょう）されること	大切でない	大切である:	そう感じない	あまりそう感じない	どちらでもない	ややそう感じる	そう感じる
13　自分のこころの健康面での体験を理解すること	大切でない	大切である:	そう感じない	あまりそう感じない	どちらでもない	ややそう感じる	そう感じる
14　自分にとって意味のあることをすること	大切でない	大切である:	そう感じない	あまりそう感じない	どちらでもない	ややそう感じる	そう感じる
15　困難を経験した自分の生活を立て直すこと	大切でない	大切である:	そう感じない	あまりそう感じない	どちらでもない	ややそう感じる	そう感じる
16　満足のいく暮らしを送ること	大切でない	大切である:	そう感じない	あまりそう感じない	どちらでもない	ややそう感じる	そう感じる
17　自分の生活を自分で決めていると感じること	大切でない	大切である:	そう感じない	あまりそう感じない	どちらでもない	ややそう感じる	そう感じる

サポート（支援）

私のリカバリーにとって... / **担当スタッフから支援されていると感じますか**

18　こころの健康を自己管理できること	大切でない	大切である:	そう感じない	あまり そう感じない	どちら でもない	やや そう感じる	そう感じる
19　新しいことをやってみること	大切でない	大切である:	そう感じない	あまり そう感じない	どちら でもない	やや そう感じる	そう感じる
20　強み・長所を伸ばしていくこと	大切でない	大切である:	そう感じない	あまり そう感じない	どちら でもない	やや そう感じる	そう感じる

関係性

あなたとあなたの担当スタッフとの間の関係について、もっともあてはまるものに〇をつけてください。

1	私は、担当スタッフに自分の話を聞いてもらえていると感じる	そう思わない	あまり そう思わない	どちら でもない	ややそう思う	そう思う
2	私は、担当スタッフに支えられていると感じる	そう思わない	あまり そう思わない	どちら でもない	ややそう思う	そう思う
3	担当スタッフは、私の夢や希望を真剣に受け止めていると感じる	そう思わない	あまり そう思わない	どちら でもない	ややそう思う	そう思う
4	担当スタッフは、私のことを尊重(そんちょう)している	そう思わない	あまり そう思わない	どちら でもない	ややそう思う	そう思う
5	担当スタッフは、一個人として私に接している（診断名やレッテルを持った人としてではなく）	そう思わない	あまり そう思わない	どちら でもない	ややそう思う	そう思う
6	担当スタッフは、私のことは私自身が決めるようにサポートしている	そう思わない	あまり そう思わない	どちら でもない	ややそう思う	そう思う
7	たとえ私がどん底に落ち込んでいたとしても、担当スタッフは私に希望を持ったままでいてくれる	そう思わない	あまり そう思わない	どちら でもない	ややそう思う	そう思う

最後までご回答いただき
ありがとうございました。

INSPIRE は Julie Williams, Mary Leamy, Mike Slade とロンドン大学キングス・カレッジの同僚らによって英語で開発されました。詳細な情報は、researchintorecovery.com/inspire から入手できます。
日本語版 INSPIRE は東京大学チームによって翻訳されました http://plaza.umin.ac.jp/heart/archives/inspire.shtml
INSPIRE was developed in English by Julie Williams, Mary Leamy, Mike Slade and colleagues at King's College London.
Further information available from researchintorecovery.com/inspire. This version was translated into Japanese by the University of Tokyo team.
