# Supplementary material for: Reliability and validity of the Japanese version of the INSPIRE measure of staff support for personal recovery in community mental health service users in Japan
Source: BMC Psychiatry. 2020 Feb 7;20:51. doi: 10.1186/s12888-020-2467-y (PMC7006071; doi:10.1186/s12888-020-2467-y)
Supplement: Supplementary file 2 — Additional file 2. Japanese version of Brief INSPIRE. [file 12888_2020_2467_MOESM2_ESM.pdf]

# INSPIRE

## 短縮版

「リカバリー」は色々な意味として語られ、そのひとつは、「満足いく、希望に満ちた人生をおくること」です。
この質問は、担当スタッフが、あなたのリカバリーをどのように支えているかをお聞きします。
（担当のスタッフがいない場合は、あなたがもっともよく接するスタッフ、あるいはよく相談をするスタッフをひとり思い浮かべてお答えください。）

＿＿＿＿＿＿＿＿＿＿＿＿についてすべての質問にお答えください。
（担当スタッフの名前）

担当スタッフから、あなたのリカバリーをどれくらい支援されていると感じますか、もっともあてはまるところに○をつけてください。

| | | | | | | |
|---|---|---|---|---|---|---|
| 1 | 担当スタッフは、私がまわりの人からサポートを受けていると感じられるように支援してくれる | そう思わない | あまりそう思わない | どちらでもない | ややそう思う | そう思う |
| 2 | 担当スタッフは、私が将来に夢や希望をもつことを支援してくれる | そう思わない | あまりそう思わない | どちらでもない | ややそう思う | そう思う |
| 3 | 担当スタッフは、私が自分自身をよく思えるように支援してくれる | そう思わない | あまりそう思わない | どちらでもない | ややそう思う | そう思う |
| 4 | 担当スタッフは、私が自分にとって意味のあることをすることを支援してくれる | そう思わない | あまりそう思わない | どちらでもない | ややそう思う | そう思う |
| 5 | 担当スタッフは、私が自分の生活を自分で決めていると感じられるように支援してくれる | そう思わない | あまりそう思わない | どちらでもない | ややそう思う | そう思う |

INSPIRE は Julie Williams, Mary Leamy, Mike Slade とロンドン大学キングス・カレッジの同僚らによって英語で開発されました。詳細な情報は、researchintorecovery.com/inspire から入手できます。
日本語版 INSPIRE は東京大学チームによって翻訳されました http://plaza.umin.ac.jp/heart/archives/inspire.shtml
INSPIRE was developed in English by Julie Williams, Mary Leamy, Mike Slade and colleagues at King's College London.
Further information available from researchintorecovery.com/inspire. This version was translated into Japanese by the University of Tokyo team.
